# Novel high-yield potato protease inhibitor panels block a wide array of proteases involved in viral infection and crucial tissue damage

**DOI:** 10.1007/s00109-024-02423-x

**Published:** 2024-02-21

**Authors:** Nienke Visser, Laure C. M. Herreman, Jennifer Vandooren, Rafaela Vaz Sousa Pereira, Ghislain Opdenakker, Robin E. J. Spelbrink, Maarten H. Wilbrink, Edwin Bremer, Reinoud Gosens, Martijn C. Nawijn, Heidi H. van der Ende-Metselaar, Jolanda M. Smit, Marc C. Laus, Jon D. Laman

**Affiliations:** 1 Department of Hematology, Cancer Research Center Groningen, University Medical Center Groningen, University of Groningen, 9713 GZ Groningen, The Netherlands; 2 Avebe Innovation Center Groningen, 9747 AW Groningen, The Netherlands; 3 Laboratory of Immunobiology, Rega Institute for Medical Research, Department of Microbiology, Immunology and Transplantation, KU Leuven, 3000 Louvain, Belgium; 4 Department of Molecular Pharmacology, University Medical Center Groningen, 9713 GZ Groningen, The Netherlands; 5 Groningen Research Institute for Asthma and COPD (GRIAC) Research Institute, University of Groningen, 9713 GZ Groningen, The Netherlands; 6 Department of Pathology and Medical Biology, University of Groningen, University Medical Center Groningen, 9713 GZ Groningen, The Netherlands; 7 Department of Medical Microbiology and Infection Prevention, University Medical Center Groningen, University of Groningen, 9713 GZ Groningen, The Netherlands

**Keywords:** Protease inhibitor, Potato, Covid-19, Viral infection, Tissue preteolysis

## Abstract

**Abstract:**

Viruses critically rely on various proteases to ensure host cell entry and replication. In response to viral infection, the host will induce acute tissue inflammation pulled by granulocytes. Upon hyperactivation, neutrophil granulocytes may cause undue tissue damage through proteolytic degradation of the extracellular matrix. Here, we assess the potential of protease inhibitors (PI) derived from potatoes in inhibiting viral infection and reducing tissue damage. The original full spectrum of potato PI was developed into five fractions by means of chromatography and hydrolysis. Individual fractions showed varying inhibitory efficacy towards a panel of proteases including trypsin, chymotrypsin, ACE2, elastase, and cathepsins B and L. The fractions did not interfere with SARS-CoV-2 infection of Vero E6 cells in vitro. Importantly, two of the fractions fully inhibited elastin-degrading activity of complete primary human neutrophil degranulate. These data warrant further development of potato PI fractions for biomedical purposes, including tissue damage crucial to SARS-CoV-2 pathogenesis.

**Key messages:**

- Protease inhibitor fractions from potato differentially inhibit a series of human proteases involved in viral replication and in tissue damage by overshoot inflammation.
- Protease inhibition of cell surface receptors such as ACE2 does not prevent virus infection of Vero cells in vitro.
- Protease inhibitors derived from potato can fully inhibit elastin-degrading primary human neutrophil proteases.
- Protease inhibitor fractions can be produced at high scale (hundreds of thousands of kilograms, i.e., tons) allowing economically feasible application in lower and higher income countries.

**Supplementary Information:**

The online version contains supplementary material available at 10.1007/s00109-024-02423-x.

## Introduction 

Proteases, derived from either the host or the virus itself, are essential during virus replication. Proteases have been identified to play key roles in each step of the virus replication cycle, i.e., virus receptor binding, membrane fusion, protein translation and folding, genome replication, and assembly of new virus particles. Which proteases are required is, however, virus specific [1–4]. For example, positive-sense RNA viruses encode a polyprotein that must be proteolytically processed into protein fragments to ensure progeny virion formation and spreading. Viral proteins are classified as structural proteins, proteins that are part of the physical structure of the virion, or non-structural proteins which are not part of the structure of the virion and essential for the replication of the genome. Viral proteins are also known to inhibit or promote cellular pathways to ensure efficient viral genome replication, e.g., shutdown of host cell protein synthesis. Proteolytic cleavage of the polyprotein into protein fragments is mediated through host and viral proteases. Among the viral proteases involved, cysteine proteases (adenovirus, alphavirus, picornavirus, coronavirus), serine proteases (flavivirus, coronavirus), and aspartic acid proteases (HIV) have been extensively characterized [5–9] as potential targets for antiviral drugs. Several viruses also employ host proteases for binding and activation of viral proteins before membrane fusion, notably the serine transmembrane protease serine 2 (TMPRSS2) used by influenza and coronaviruses, or for posttranslational cleavage of the viral polyprotein, such as the cell furin-like protease used by influenza and alphaviruses [10, 11]. In the case of coronaviruses (SARS-CoV, SARS-CoV-2, and MERS-CoV), viral surface spike protein S mediates cell entry through the binding to a cell surface receptor (e.g., ACE2 or DPP4) [12]. The spike protein S is then cleaved by host proteases such as TMPRSS2 or furin allowing membrane fusion [13]. Neighboring serine proteases such as trypsin, tryptase, or plasmin are also suspected to promote S protein cleavage and enhance virus entry into host cell [10, 14, 15].

As dramatically underscored by the recent SARS-CoV-2 pandemic, some viruses can lead to severe inflammation. Principally located in the airway tissues, SARS-CoV-2-induced inflammation may, however, develop in other organs and become life-threatening for the host [16]. Tissue inflammation is characterized by an excessive infiltration and activation of blood leukocytes. Innate immune cells such as neutrophils and macrophages constitute the first lines of host defense to viral infection. In addition of being the most abundant leukocyte type in a normal host (about 70% of circulating leukocytes), increased levels of circulating neutrophils have especially been reported in severely affected SARS-CoV-2 cases [17]. Whereas neutrophils can prevent virus spreading through phagocytosis, degranulation of virus-killing proteases, or virus trapped into neutrophil extracellular traps (NET), an overwhelming neutrophil infiltration may lead to tissue injury due to excessive oxidative damage and extracellular matrix proteolysis. Dysfunctional neutrophil activation may also contribute to the viral spread itself through the cleaving the spike S protein by neutrophil elastase (NE) [18]. Moreover, aberrant NET formation observed in severe cases of SARS-CoV-2 is suspected to interfere with host immune responses through downregulation of genes involved in their interaction with T and NK cells, leading to insufficient antiviral response [19].

The characterization of individual and panels of protease inhibitors (PI) is one of the strategies to target viral infection and related tissue injury. PI can be isolated from different natural sources, such as soybean and potatoes. One of the protein fractions of industrial starch potatoes consists of over 21 well-characterized PI [20]. These protease inhibitors are produced by the plant to defend the tuber against pathogens and herbivore attack. The PI derived from potatoes are naturally present in the seeds and tubers and are the most abundant group of proteins present into the so-called potato fruit juice, the juice obtained after removal of the starch. Potato PI can be classified into seven subgroups, based on their enzyme-inhibiting activities and intrinsic characteristics (molecular weight, isoelectric point) [20]. Plant protease inhibitors in general and potato protease inhibitors specifically have been reported to possess interesting health-promoting biologic activities [21]. We have previously demonstrated that this panel effectively limits skin inflammation in stoma patients and infants with congenital defects of the innervation of the colon, since they inhibit the spectrum of gut proteolytic enzymes [22]. In general, major advantages of potato protease inhibitors in terms of regulation and patient/customer acceptance include FDA approval as Generally Recognized As Safe (GRAS) [23], absence of animal material (including eg protein), and absence of allergens and genetically modified organisms (GMO).

In this study, employing novel fractions of potato protease inhibitors, we tested the hypotheses that:

1. SARS-CoV-2 infection and replication can be inhibited since the infection/replication cycle critically depends on proteases, from both the virus and the human host.
2. Proteolytic enzymes released by host white blood cells (notably granulocytes and macrophages) can be inhibited, thereby reducing inflammatory tissue dysfunction.

We argue that the findings of the current study warrant further development of protease inhibitor fractions for biomedical purposes, including viral infections with hyperinflammation, as observed in COVID-19.

## Results

### Potato protease inhibitor fractions vary in composition

**Fig. 1:**
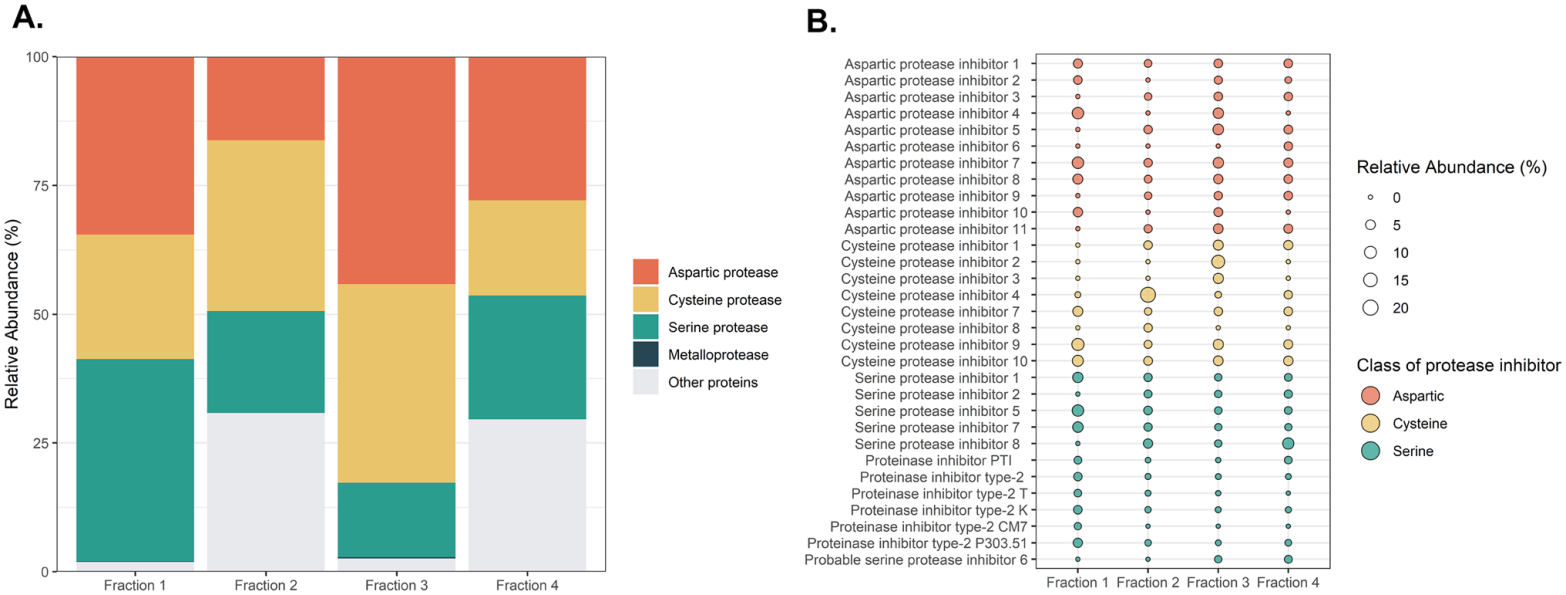
Fractions vary in protease inhibitor composition. **A** Relative abundance of class of protease inhibitors within each fraction. **B** Detailed composition of protease inhibitors. Six protease inhibitors with relative abundance lower than 1.2% in all four fractions are not displayed in **B**. Relative abundance expressed as percentage of total protein within the fraction. N.B. Fraction 5 is prepared from the same starting material as fraction 4; hence, proteomics analysis of fraction 5 was not performed

For this study, a major fraction of potato protease inhibitors available at industrial scale from potato fruit juice was used for further separation into five different fractions. One fraction resulted from the soluble part after pH adjustment. Two fractions were obtained by means of chromatographic separation, and two fractions were generated by proteolytic hydrolysis to obtain peptides derived from PI. Building on the work of Pouvreau et al. [24] that identified and characterized 21 PI from potato fruit juice, state of the art proteomics analysis identified 37 PI (Fig. 1). In this pool of PI, 16 serine PI, 11 aspartate PI, nine cysteine PI, and one metallo PI were detected throughout the different fractions.

Fraction 1 included a majority of aspartic acid and serine PI, fraction 2 contained a large part of cysteine proteases, while fraction 3 presented dominant cysteine and aspartate PI portions (Fig. 1A). In these last two fractions, the composition of cysteine protease inhibitors varied with prevailing cysteine PI 4 (19.3% relative protein abundance (RPA)) in fraction 2 and cysteine PI 2 (12.9% RPA) in fraction 3 (Fig. 1B). As anticipated, metalloproteinase inhibitors were nearly absent in all fractions (0.13% and 0.26% RPA of a metallocarboxypeptidase inhibitor in fraction 1 and fraction 3, respectively). In addition to protease inhibitors, other proteins, such as glycolytic and chitinolytic enzymes, lipoxygenase, or patatin, were also detected in the different fractions. These non-PI compounds were present in greater amount in fractions 2 and 4 (30.8% and 29.6% RPA, respectively).

### Potato PI fractions inhibit a range of proteases

**Fig. 2:**
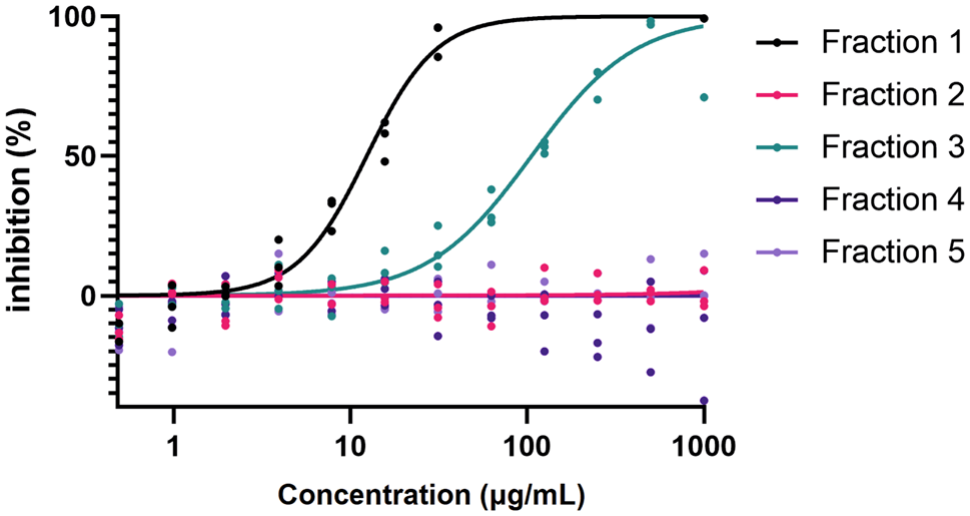
Inhibition of potato PI fraction 1 and 3 on elastase. Dose-dependent response for the inhibition or elastase of elastin by fraction 1 and 3 (*n* = 3)

**Fig. 3:**
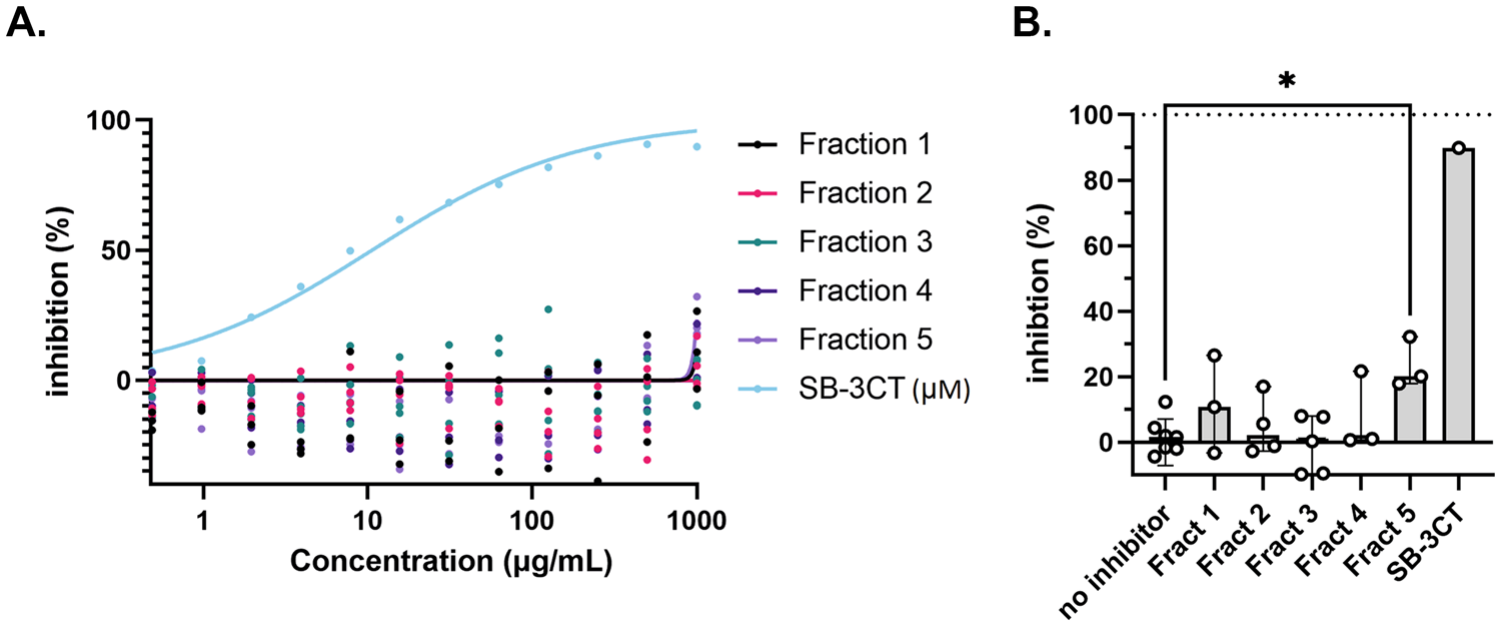
Fractions do not inhibit OmniMMP proteolysis by MMP-9. **A** Dose-response for the inhibition of MMP-9 activity (0.1 nM) by fractions 1–5 and SB-3CT. **B** Inhibition of MMP9 catalysis by the fractions at a concentration of 1 mg/mL. *N* ≥ 3, behalf SB-3CT, *N* =1. *p < 0.05. Bars represent median values, and error bars represent the 95% confidence interval. Statistical differences were tested by Kruskal–Wallis test with Dunn’s multiple comparisons test

The inhibitory activities of the five fractions were assessed via distinct protease activity assays against a panel of proteolytic enzymes relevant to virus infection and tissue damage. Fraction 1 was the most effective in inhibiting serine proteases as shown by low IC_50_ values for trypsin, chymotrypsin, and elastase (Table 1). Complementary analysis using an elastin substrate showed a clear dose-dependent inhibition of elastase activity by both fractions 1 and 3 (Fig. 2; Table 2). Fractions 2 and 3 were the most effective in inhibiting cysteine protease cathepsin B and metallocarboxypeptidase ACE2, whereas fractions 1, 3, 4, and 5 effectively inhibited cathepsin L activity. None of the fractions inhibited furin and collagenase activities (Table 1). To determine whether the fractions inhibited MMP-9, a major enzyme in tissue damage, a dose-response curve for each fraction was generated (Fig. 3). Due to the low inhibition of MMP-9, no reliable calculation of IC_50_ was possible (Table 2). At the highest concentration, 1 mg/mL, fraction 5 gave a significant but modest inhibition of MMP-9 of 20%.

**Table 1:** Potato PI fractions inhibit a range of proteases relevant to virus infection and tissue damage

Protease	Cysteine			Metallo-			Serine			
	CatB	CatL	Papain	Collagenase	ACE1	ACE2	Trypsin	Chymotrypsin	Human leukocyte elastase	Furin
Sample	IC_50_ (mg protein/L)									
Fraction 1	nd	< 25	6.419	nd	1129	> 1000	33	73	79	nd
Fraction 2	5.2	> 250	254	nd	61	29	1.439	296	nd	nd
Fraction 3	59	< 25	453	nd	717	32	58	159	nd	nd
Fraction 4	> 500	< 25	> 625	nd	182	255	nd	nd	nd	nd
Fraction 5	> 500	< 25	> 625	nd	344	250	nd	nd	nd	nd

Values “larger than”: inhibition occurred, but the highest dose tested failed to inhibit 50% of the proteolytic activity. Values “smaller than”: the lowest dose tested resulted in near-complete inhibition. *n* = 1

*nd* no inhibitory activity detected, *CatB* Cathepsin B, *CatL* Cathepsin L, *ACE1* Angiotensin I Converting Enzyme, *ACE2* Angiotensin Converting Enzyme 2, *IC50* half-maximal inhibitory concentration

**Table 2:** Comparative inhibition of elastase and MMP-9 by potato fractions on elastin and OmniMMP substrate, respectively

	**Elastase (on elastin)**	**MMP-9 (on OmniMMP)**
**Sample**	**IC**_**50**_** (μg/mL)**	
**Fraction 1**	12.34	N/A
**Fraction 2**	N/A	N/A
**Fraction 3**	105.8	N/A
**Fraction 4**	N/A	N/A
**Fraction 5**	N/A	N/A
**Halt inhibitors**^**a**^	NM, 94% inhibition at 1:100 dilution	NM
**SB-3CT**	NM	10.61 μM
**EDTA**	NM	NM, 100% inhibition at 100 nM

*IC*_*50*_ half-maximal inhibitory concentration, *N/A* no data available due to out of range, low accuracy, or no inhibition, *NM* not measured

^a^Only one concentration tested

**Fig. 4:**
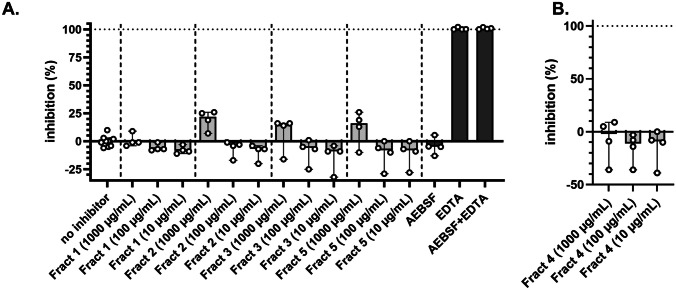
Minimal inhibition of fractions 1–5 on the proteolysis of OmniMMP substrate by neutrophil proteases. **A** Inhibition of neutrophil proteases by four fractions and selected inhibitors. **B** Inherent proteolytic activity in fraction 4. EDTA, metalloprotease inhibitor; AEBSF, serine protease inhibitor. Bars represent median values and error bars represent the 95% confidence interval

**Fig. 5:**
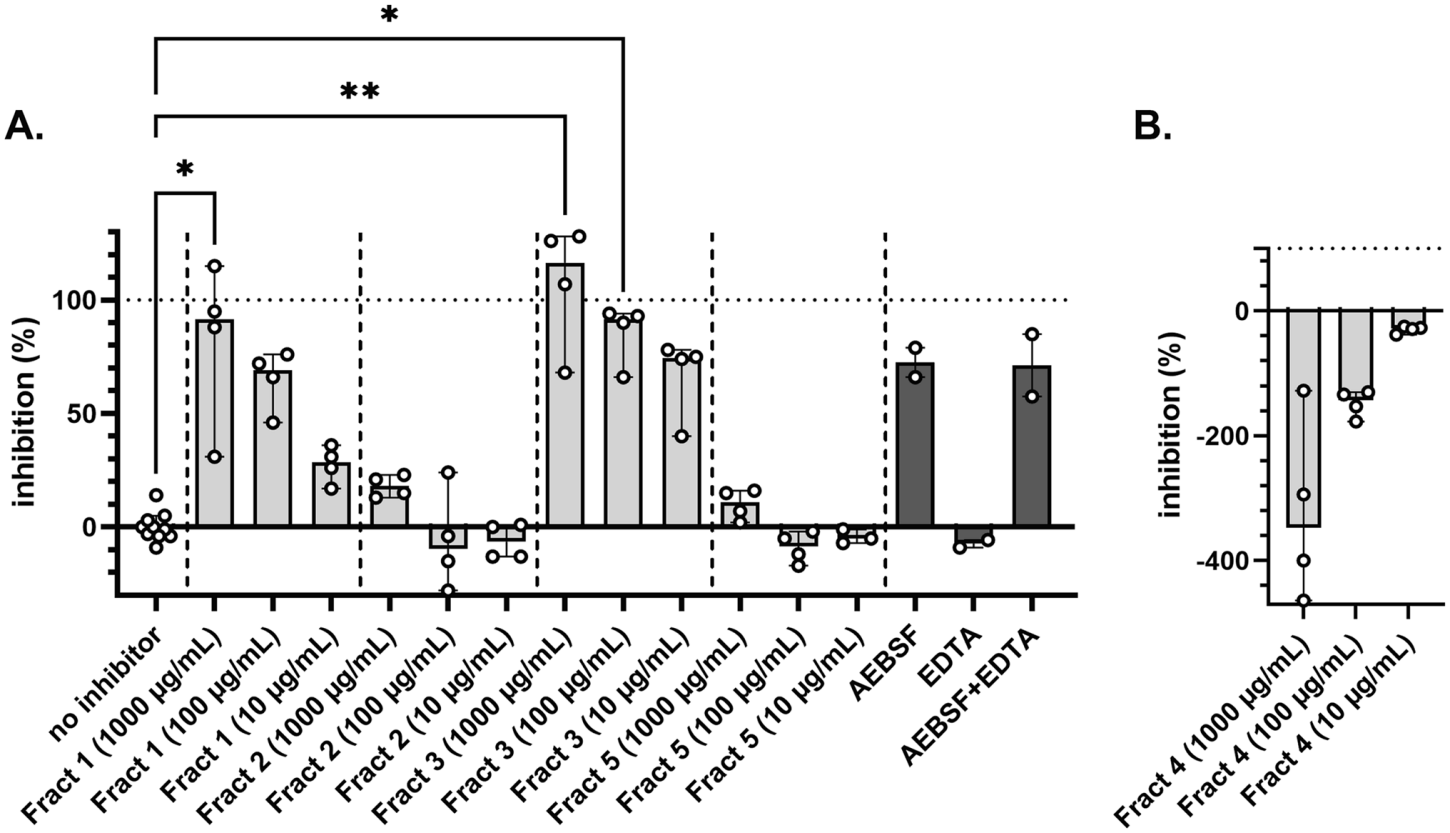
Fractions 1 and 3 effectively inhibit the proteolysis of elastin by neutrophil proteases. **A** Inhibition of neutrophil proteases by four fractions and selected inhibitors. **B** Intrinsic proteolytic activity in fraction 4. EDTA, metalloprotease inhibitor; AEBSF, serine protease inhibitor. *p < 0.05, **p < 0.01. Bars represent median values, and error bars represent the 95% confidence interval. Statistical differences were tested by Kruskal–Wallis test with Dunn’s multiple comparisons test

**Fig. 6:**
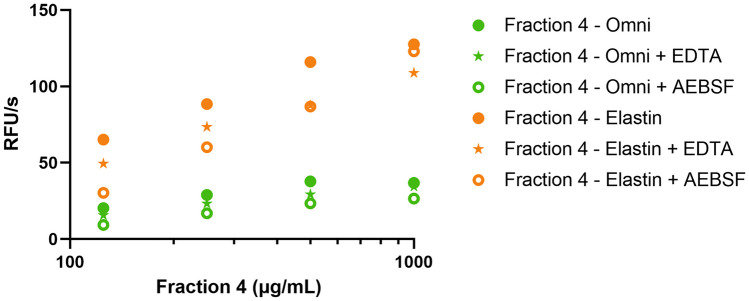
Proteolysis of elastin and OmniMMP substrate by fraction 4. EDTA, metalloprotease inhibitor; AEBSF, serine protease inhibitor

Next, protease inhibition of the fractions towards primary human neutrophil degranulate was tested on two substrates. No significant inhibition was seen of the proteolysis of the broad MMP substrate by neutrophil MMP proteases (Fig. 4A). In contrast, proteolysis of elastin was significantly inhibited by fractions 1 and 3 (Fig. 5A). This observation is in line with our analysis of elastin degradation by purified elastase (Fig. 2; Table 2), where both fraction 1 and fraction 3 provided significant inhibition. Finally, in the presence of fraction 4, proteolysis was increased, in particular for elastin (Figs. 4B and 5B). Hence, we next analyzed whether the extracts themselves had any proteolytic activity (Fig. 6). In the presence of fraction 4, but none of the other fractions, modest proteolysis of mainly the elastin substrates was observed. As expected, this activity was almost insensitive to inhibition by EDTA as a metalloprotease inhibitor or by AEBSF as a serine protease inhibitor (Fig. 6).

### Potato PI fractions affect Vero E6 cell viability to various degrees

**Fig. 7:**
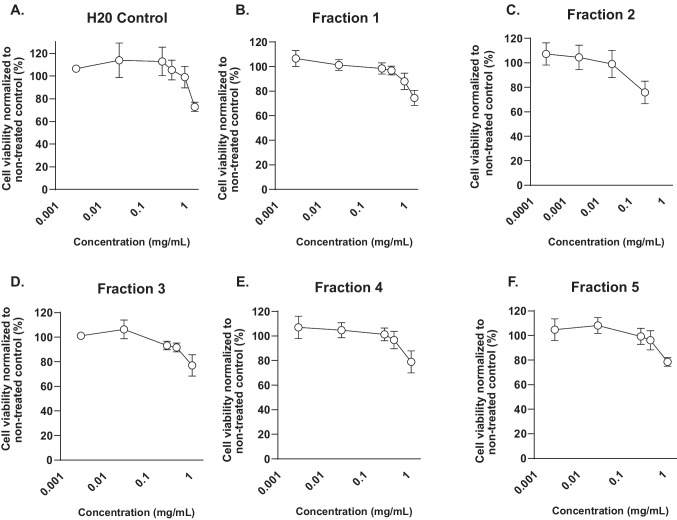
Potato PI fractions affect viability of Vero E6 cells. **A** Cell viability of Vero E6 cells treated with H20 control. **B**–**F** Viability of Vero E6 cells treated with different concentrations of PI fractions 1–5. Data are represented as dose-response curve with mean ± SEM from three independent experiments. All individual experiments were performed in triplicate

To determine the effect of potato PI on cell viability, Vero E6 cells were treated with increasing concentrations of each PI fraction for 24 h. As illustrated in Fig. 7, the concentration range leading to minimal loss in cell viability differed between PI fractions. Vero E6 cells exposed to 0.5 mg/mL of fractions 1, 3, 4, and 5 maintained a cell viability around 90–100%. In contrast, fraction 2 was more toxic for the cells, the highest non-toxic concentration was 0.03 mg/mL.

### Protease inhibitor fractions do not limit in vitro infection of Vero cells by SARS-CoV-2

**Fig. 8:**
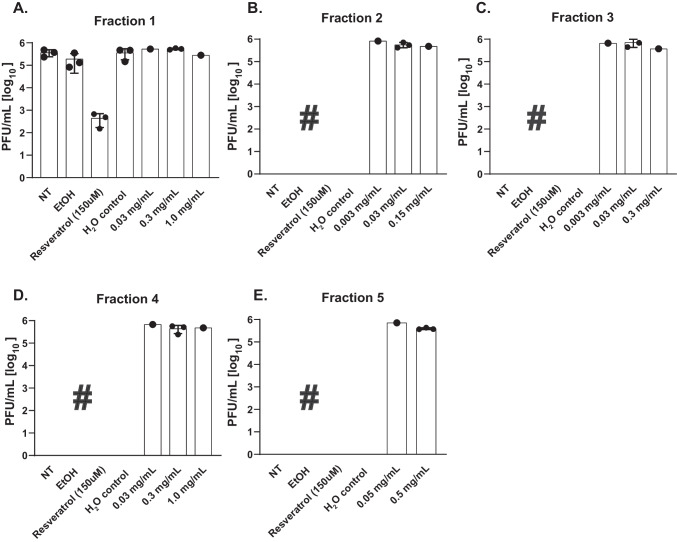
No prevention of SARS-CoV-2 infection of Vero E6 cells by the five PI fractions. **A**–**E** SARS-CoV-2 infection in combination of protease inhibitor fractions 1–5 (*N* = 3). Per experiment the controls and fractions 1–5 were tested simultaneously. NT, not treated; EtOH, ethanol control (vehicle of resveratrol); Resveratrol, positive control; H_2_O control, water control (vehicle of PI)

To assess whether PI fractions interfered with SARS-CoV-2 infection or virus production, Vero E6 cells were pre-treated with increasing concentrations of the PI fractions and were infected with SARS-CoV-2. The concentrations tested were based on the cell viability results and the amount of compound available. Progeny virus production was determined by plaque assay at 8 h post-infection (hpi). Resveratrol (diluted in EtOH) was used as a positive control [25]. None of the five protease inhibitor fractions interfered with in vitro infection of SARS-CoV-2 in Vero E6 cells (Fig. 8) at the concentrations tested.

### Fraction 3 inhibits in vitro ACE2 activity in Vero E6 cells

**Fig. 9:**
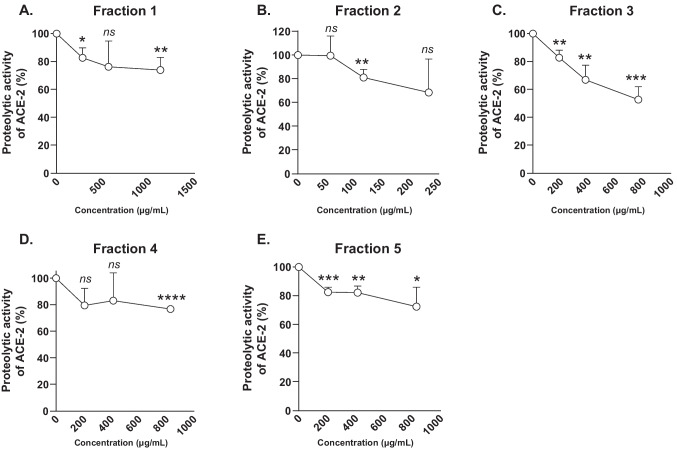
Inhibition of ACE2 activity in Vero E6 cells by the PI fractions. **A**–**E** Proteolytic activity of ACE2 measured from fractions 1–5 in Vero E6 cells. All individual experiments were performed in triplicate. Significance was tested using Students’ test. *p* values are indicated as ****p < 0.0001, ***p < 0.001, **p < 0.01, and *p < 0.05

Next, the in vitro proteolytic activity of ACE2 in Vero E6 cells exposed to the five fractions was determined. All fractions showed some inhibition of ACE2 proteolytic activity (Fig. 9). Fractions 1, 4, and 5 showed a mean maximal inhibitory effect of 26%, 23%, and 28%, respectively, whereas fraction 3 displayed a much higher ACE2 inhibition at the highest concentration tested (800 μg/mL), with a mean inhibition 52.8%. Following the cell viability assay, fraction 2 was tested at a lower concentration range. At 125 μg/mL, this fraction significantly inhibited 20% of ACE2 activity. Albeit not significant, a decreasing trend was further observed at higher concentration tested (240 μg/mL). This in vitro data set is line with the results of ACE2 protease activity assay presented above (Table 1).

### Airway single-cell RNAseq data of proteases involved in viral infection and tissue damage

**Fig. 10:**
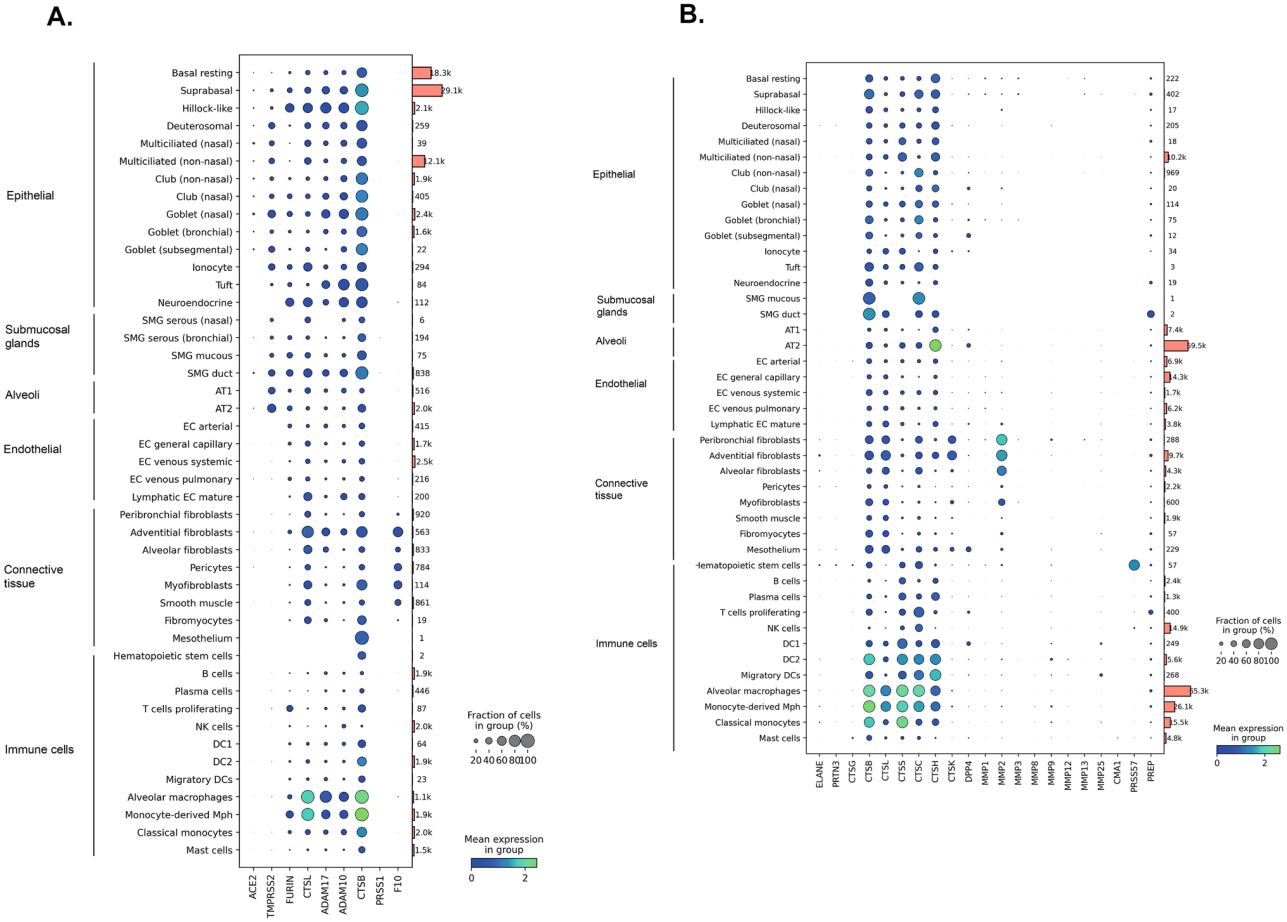
Single RNA sequencing of airway cell subsets: proteases involved in viral infection and tissue damage. **A** Dot plot of marker genes of host proteases involved in SARS-CoV-2 cell entry replication in cell subsets from airway epithelium. **B** Host proteases involved in lung tissue damage in cell subsets from the parenchyma. The marker genes are represented in columns, and cell types are in rows. The orange bars indicate the number of cells of the given cell type. DC, dendritic cells; NK, natural killer; Mph, macrophages; EC, endothelial cells; AT, alveolar type; SMG, submucosal glands

For potential aerosol administration of protease inhibitors, it is crucial to assess which proteases are expressed by what cell types, and at what level of the bronchial tree and lungs. Therefore, expression of protease genes involved in SARS-CoV-2 infection and tissue damage in airways was determined from available single-cell RNA sequencing of the Single Cell Atlas (SCA) consortium (data based on healthy controls). ACE2 and TMPRSS2 were mostly expressed in epithelial, submucosal glands (SMG), and alveoli cells. Cathepsin L and cathepsin B RNA were expressed in most cell subsets. More specifically, cathepsin L showed expression in a greater fraction of hillock-like cells, adventitial fibroblasts, alveolar macrophages, and monocyte-derived macrophages. Cathepsin B displayed a greater mean RNA expression in numerous epithelial cell subsets (suprabasal, hillock-like, club and goblet cells), SMG ducts, alveolar macrophages, and monocyte-derived macrophages (Fig. 10A). Among the proteases potentially involved in lung tissue damage, cysteine lysosomal proteases (cathepsins B, L, S, C, H, K) were ubiquitously expressed in airway cells, with higher expression in innate immune cell types, SMG subgroups (cathepsins B and C), or alveoli type 2 cells (cathepsin H) (Fig. 10B). Of the MMPs, MMP-2 RNA was mostly present in fibroblasts, while MMP-9 was only expressed in minor fractions of peribronchial fibroblasts, dendritic cells type 2, and monocyte-derived macrophages. Neutrophil serine proteases such as elastase, proteinase 3, and cathepsin G were largely absent in this healthy control dataset.

## Discussion

This study assessed whether differentially fractionated potato protease inhibitors can limit the SARS-CoV-2 replication cycle in vitro, and whether these fractions inhibit the activity of a series of white blood cell enzymes that mediate tissue damage in a wide range of inflammatory diseases.

Apart from furin, MMP-9, and collagenase, most of the 11 selected proteases were inhibited by one or multiple PI fractions in protease activity assays (Table 3). However, the various PI fractions did not prevent SARS-CoV-2 infection of Vero E6 cells in vitro.

**Table 3:** Inhibitory activity of potato PI fractions against enzymes in viral replication and tissue damage

Protease	Cysteine			Metallo-				Serine					Serine and metallo
	CatB	CatL	Papain	Collagenase	ACE1	ACE2	Gelatinase (MMP-9)	Trypsin	Chymotrypsin	Furin	Porcine pancreas elastase	Human leukocyte elastase	Human neutrophil degranulate
Fraction 1	-	+++	+	-	+	+	-	+++	+++	-	+++	+++	+++
Fraction 2	+++	+	++	-	+++	+++	-	+	++	-	-	-	-
Fraction 3	+++	+++	++	-	+	+++	-	+++	++	-	++	-	++++
Fraction 4	+	+++	+	-	++	++	-	-	-	-	-	-	-
Fraction 5	+	+++	+	-	+	++	+	-	-	-	-	-	-

+++: high inhibitory activity, ++: moderate inhibitory activity, +: low inhibitory activity, -: no inhibitory activity

### Inhibition of proteases involved in SARS-CoV-2 virus entry and replication

Based on single-cell RNA seq data from healthy controls, ACE2 and TMPRSS2 were mostly expressed in epithelial and alveolar cell subsets, in agreement with previous datasets. This specific location at the air-tissue interface exposes these host proteases directly to the respiratory viruses, enabling specific virus cell attachment and activation. Upon SARS-CoV-2 infection, epithelial secretory cells, FOXN4 + differentiating cells, and ciliated cells show the greatest increase in ACE2 RNA expression relative to healthy controls [26]. Concomitant expression of ACE2, TMPRSS2, and/or furin was especially increased in these cell subsets, suggesting a higher susceptibility for viral infection [26].

Fractions 2 and 3 were the most effective in reducing the protease activity of ACE2 in Vero E6 cells, yet these fractions did not limit SARS-CoV-2 cell infection in vitro. This suggests that the specific binding site of potato PI to ACE2, leading to its proteolytic inactivation, does not impair the ACE2-spike S-glycoprotein interaction. While the Vero E6 cell line highly expresses the ACE2 receptor, it does not express the trypsin-like TMPRSS2 [27]. Consequently, any potential inhibitory activity of potato serine PI towards TMPRSS2 could not prevail in the current experimental setup. This is in line with previous studies, where serine protease inhibitors such as aprotinin and camostat mesylate were ineffective in blocking SARS-CoV-2 infection in the Vero E6 cell line, but were highly effective in cells expressing TMPRSS2 [28–30]. In this Vero E6 cell model, SARS-CoV-2 infects cells via endocytosis and its activation relies on intracellular acid-activated (pH 4.5–5.0) lysosomal protease cathepsin L [28]. Four of the 5 PI fractions showed high inhibition of cathepsin L activity (IC_50_ < 25 mg protein/L; Tables 1 and 3). However, the same fractions failed to interfere with viral infection into the cells. Similarly, the well-established inhibitory effect of potato PI on papain and chymotrypsin activity [24, 31, 32] did not interfere with in vitro viral papain-like (PLpro) and chymotrypsin-like viral proteases (3CLpro or Mpro) involved in virus replication. However, other cathepsin L inhibitors prevented infection of cells lacking TMPRSS2 when those were incubated with inhibitors prior to SARS-CoV-2 infection [28, 29, 33, 34]. Successfully tested cathepsin L inhibitors, such as E-64d, SID26681509, SB412515, or amantadine, are small molecules ranging from 188 to 540 Da, whereas potato PI molecular weights mostly vary from 4.3 to 45 kDa. Probably, potato PI remained in the extracellular space unable to interact with the intracellular proteases. Potato PI are also known to preserve their biological activity at low pH (from pH 3) [24]; therefore, the acidic environment of the lysosome is unlikely to be the cause of their ineffectiveness. Another plausible explanation lies in the concentration range of PI fractions used during and after infection of the Vero E6 cell line: the concentration of cathepsin L inhibitors — selected following the cell viability tests — was too low to neutralize cathepsin L protease activity. Owing to the particular endosomal entry route of the virus in Vero E6 cells, cathepsin L is indeed highly expressed in comparison with other lines such as Calu-3 [29], therefore requiring higher concentrations of inhibitors.

### Inhibition of proteases involved in tissue damage such as the lungs

Neutrophils are the most abundant circulating leukocytes and are considered the first line of defense of the immune system against infection. Upon SARS-CoV-2 infection, increased expression levels and hyperactivation of neutrophils occur in upper and lower respiratory tracts and correlate with disease severity [26, 35, 36]. Neutrophil proteases are pro-inflammatory when a perpetuating neutrophil infiltration occurs concomitantly with an inadequate protease inhibition, leading to excessive tissue damage. Fraction 1 represents a “total” extract of the potato tubers’ arsenal of protease inhibitors soluble at pH 8.0 and is mostly proteinaceous in nature (*N* * 6.25 > 90% on dry matter). Its resulting subfractions (fractions 2 and 3) are characterized by specific serine, cysteine, and aspartic acid protease inhibitors (detailed in Supplementary Table S1). Multiple potato serine protease inhibitors, including protease inhibitor type 2, as well as potato cysteine protease inhibitors inhibit human or porcine elastase [24, 38, 39]. Two of the five potato PI fractions effectively inhibited elastin-degrading neutrophil proteases. Of the neutrophil proteases, elastase could be effectively inhibited by fractions 1 and 3. Interestingly, fraction 3 inhibited human neutrophil degranulate, but did not inhibit elastase as shown in Table 3. Most likely another serine protease (trypsin-like) is responsible for this, since fraction 3 can inhibit trypsin and chymotrypsin. It was previously described that fMLF-stimulated neutrophils also secrete proteinase 3 and cathepsin G [37]. The effect of potato protease inhibitors (as a whole or divided into fractions) towards neutrophil degranulate has not been tested before. The present study therefore reveals their inhibitory power towards proteinase 3 and cathepsin G, in addition to that of elastase. Matrix metalloproteinase 9 (MMP-9) is also known as gelatinase B. Together with the action of collagenases (MMP-1, MMP-8, and MMP-13), this enzyme is involved in the degradation of extracellular matrix components, including collagens, gelatins, laminins, fibronectin chains, and proteoglycans [40]. Potato PI did not inhibit MMP-9 nor collagenase activity, which is in line with the composition of potato PI composition established in the present study, mostly lacking metalloproteinase inhibitors. Through its inhibitory effect on other neutrophil proteases, potato PI as therapeutics may potentially contribute to breaking the vicious cycle of inflammation taking place in severe COVID-19 cases. Moreover, local PI application in the upper respiratory tract in the form of spray of cream could limit the neutrophil-mediated damage to nasal cells exposed to SARS-CoV-2, and potentially reduce the risk of long-term olfactory dysfunction [41].

### Potatoes as natural source of protease inhibitors

Potato juice is a by-product from the potato starch industry and typically contains in the range of 1–2% proteins. In total, the production of potato proteins by the starch industry in Europe is estimated between 60 and 150 kt [42, 43]. More recently, mild fractionation techniques have come in use to recover and separate the different classes of storage potato proteins on an industrial scale at near-quantitative yield. Protein recoveries of above 90% are routinely achieved (data not shown). This contrasts sharply with the manufacture of typical non-peptide protease inhibitor drugs like Camostat and Nafamostat; these are prepared in batches of 1 to 25 g from aromatic precursors via multistep organic syntheses with extraction, washing, and/or distillation steps, often at yields below 50% [44–46]. Cell fermentation is also used to produce peptide-based protease inhibitors like aprotinin, resulting in a yield of 3–4 mg/L of protease inhibitor [47, 48]. With the use of the mild fractionation techniques, the functional properties and biologic activities of the potato proteins are retained and allow the use in food, cosmetic, and pharmaceutical applications. These fractions are of high yield in absolute terms, while naturally combining multiple types of protease inhibitors. Such properties make them interesting alternative candidates to clinical grade synthetic drugs; however, further additional experiments are needed to test the possibility of using the described PI for clinical purposes. The different fractions obtained in this study display varying effects on protease inhibition, as well as cell viability, underscoring the potential of biochemical treatments and fractionation to obtain distinct biological impacts. Further characterization is needed to test their therapeutic potential to start with relevant in vivo concentrations.

## Limitations of the study

The present findings highlight the potential of natural potato protease inhibitors in reducing SARS-CoV-2 infection and tissue damage due to excess host protease activity, such as in the lungs of COVID-19 patients. Kidney epithelial Vero E6 cells derived from African green monkey are permissive cells commonly used in viral replication studies. However, this model appeared unsuitable to study the inhibition SARS-CoV-2 cell entry due to the lack TMPRSS2 commonly expressed in lung cells. Primary human cells, tissue cultures, or an in vivo model displaying features of nasal epithelium and deeper parts of the airway’s human lung characteristics, such as Calu-3 cells or human primary bronchial epithelial cells, will be more suitable to assess the inhibitory potential of potato PI to its full extent in future work. Moreover, the present cell-based infection model lacks immune system components. During an infection, proteases will be secreted by activated neutrophils and macrophages in the environment (e.g., NE, MMPs) which can contribute to viral entry or modulate the infected cells. Finally, potato PI applied in vitro will have ready access to cell surface proteases involved in viral attachment and infection, but likely do not reach intracellular compartments at sufficient levels to interfere with the virus life cycle. Hence, in the context of inhibiting the SARS-CoV-2 replication cycle using the protease inhibitors, further research is required.

## Conclusion

The present study provides in vitro evidence that potato protein inhibitor fractions with distinct biological properties produced at high scale (tons) can potentially reduce tissue damage caused by proteolysis in a wide range of inflammatory diseases.

This first proof of concept study showed the potential role of allowing the production of such fractions for clinical purposes. In the future, after thorough research on the mode of action and safety of administration using appropriate toxicology testing, this tool might be valuable for large patient groups. The potato protein inhibitors will be available at reasonable cost, since the production of these inhibitors are at high scale. In line with this, it is known that effective in vivo use of the full spectrum of 37 protease inhibitors in an over-the-counter cream against skin inflammation has been a reality for patients for several years, supporting safe use in other applications.

## Material and Methods

### PI fractionation

Intact potato PI were separated by anion-exchange chromatography, according to the method as described [6]. In short, a commercial PI-enriched protein concentrate was diluted to a final concentration of 6% and the pH was adjusted to pH 8.0 with 1 M NaOH. After centrifugation (10 min, 4500* g*) to remove insoluble protein and the clear supernatant was kept as is (fraction 1) or was subjected to anion-exchange chromatography on an Äkta Pure FPLC system equipped with an XK16 column containing Q-Sepharose Fastflow resin (Column volume 24 mL). Equilibration and sample application were done at pH 8, and proteins were eluted with a linear NaCl gradient (0–250 mM). Three peaks were obtained and collected in fractions of 5 mL, which were pooled and freeze dried. The freeze-dried material was redissolved in MilliQ and desalted on 3-kDa MWCO spin filters (Macrosep^®^ Centrifugal Filters, Pall), using diafiltration with MilliQ water (fractions 2 and 3).

### Protein hydrolysis

Heat-coagulated protein was hydrolyzed using two different commercial proteases (fractions 4 and 5): Esperase^®^ 8.0 L or Protamex^®^ (Novozymes A/S, Bagsvaerd, Denmark). Incubations were performed in batch setup using 10% w/v substrate, at 60 °C in 0.1 M Tris–HCl buffer at pH 8.0, with continuous stirring with a top stirrer (150 rpm) and without pH control. The concentration of the enzymes was 0.5% (w/v) in both cases. After 2.5 h, the proteases were inactivated by heat incubation (15 min at 90 °C) and the liquid and solid fractions were separated by centrifugation (4000* g*, 15 min). The liquid phase containing the hydrolysates was decanted, filtered on Whatman paper (Grade 595) to remove residual solids, and freeze-dried. Samples were re-dissolved in MilliQ and desalted by diafiltration over 1-kDa MWCO spin filters (Macrosep^®^ Centrifugal Filters), according to supplier instructions. Protein concentrations were determined by the use of a Kjeldahl-calibrated (Nx6.25) Sprint Protein Analyzer (CEM Corporation, Matthews). Lipopolysaccharide was removed from the fractions using ε-poly-l-lysine-agarose (Pierce High-Capacity Endotoxin Removal Spin Column, 1 mL, #88,276; Thermo Fisher Scientific, Inc.), in accordance with the manufacturer’s instructions.

### Inhibition of ACE1, ACE2, cathepsin B, cathepsin L, collagenase, and furin

ACE2, cathepsin B, cathepsin L, and furin protease activities were measured with the following fluorometric kits: ACE2 Inhibitor Screening Kit (#K310 BioVision), Cathepsin L Inhibitor Screening Kit (#K161-100, BioVision), Cathepsin B Inhibitor screening kit (MAK200, Sigma-Aldrich), and Furin Protease Assay Kit (#78,040 BPS Bioscience). Shortly, potato PI fractions were diluted in phosphate-buffered saline (PBS) and added to a 96-well black well plate with four positive, two negative, and two buffer controls per plate. Activities were measured on a fluorescence plate reader (Synergy H1) at the appropriate ex/em wavelength with automatic gain adjustment over a 1-h period at 1-min intervals. The resulting enzyme activities were reported as the maximum increase in fluorescence intensity over time per well. Inhibitory activities were calculated as percentages of intensity increase over time that were lost relative to the average of the positive controls. For the different preparations, various concentrations were tested to calculate the IC_50_ value by linear regression of the residual activity against log(concentration). ACE1 and collagenase protease activities were measured with following colorimetric kits: ACE1 Inhibitor Screening Kit (K719-100 from BioVision obtained from Bio-Connect, The Netherlands) and Collagenase Activity Colorimetric Kit (MAK293 Sigma-Aldrich).

### Inhibition of elastase

Inhibition of elastase activity was determined according to two methods. First, elastase inhibition activity was measured according to Valueva et al. [49] with minor modifications. A substrate solution was prepared by dissolving 10 mg of N-succinyl-ala-ala-ala-p-nitroanilide (Sigma-Aldrich, S4760-100 mg) in 50 µL DMSO. A solution of 15 mL of a 50-mM Tris buffer (Merck 1.08382.1000) at pH 8.2 and 5 mM CaCl_2_ (Sigma-Aldrich, C3881) was preheated to 50 °C and added to the substrate solution. Elastase concentrate human neutrophil elastase (50 µL; Calbiochem, 324,681) was diluted in 10 mL of 1 mM HCl/5 mM CaCl_2_ solution to form the enzyme solution. Sample solutions of potato protein were diluted in demineralized water to such an extent that inhibitory activities between 20 and 80% were obtained. Activities were measured by introducing 25 µL of enzyme solution and 25 µL of sample into the wells of a 96-well microtiter plate. For sample blanks, demi water was added instead of enzyme. For positive controls, demi water was added instead of sample. For negative controls, 25 µL of acetic acid was added instead of sample. The reaction was started by adding 125 µL of substrate solution to the wells. Hydrolysis of the substrate by the enzyme results in the release of p-nitroanilide with a Lambda-max of 405 nm. This was measured for 300 s at 20-s intervals on a MultiSkan Go (ThermoScientific). Activities were determined by linear regression of the A405 over time. Second, elastase inhibition activity was measured with fluorogenic kit (#E12056, Invitrogen). Shortly, pig pancreas elastase (Invitrogen, cat. no. E12056) at a concentration of 1 μg/mL was incubated with different concentrations of extract (1.000–0.5 μg/mL) or a known mixture of protease inhibitors (Halt protease inhibitor mixture, # 78437, Thermofisher) and the remaining proteolytic activity was determined by measuring the catalysis of fluorogenic elastin (15 μg/mL DQ-elastin, #E12056, Invitrogen).

### Inhibition of trypsin and chymotrypsin

Trypsin inhibitory activity and chymotrypsin inhibitory activity were measured essentially as previously described [50]. Briefly, chromogenic substrates (l-BAPNA, Na-benzoyl-l-arginine-p-nitroanilide from Sigma-Aldrich (B4875), and l-PAPNA, N-succinyl-l-phenylalanine-p-nitroanilide from Sigma-Aldrich (S4760)) were hydrolyzed by either trypsin or chymotrypsin in 50 mM Tris/HCl buffer (GE PlusOne Tris) of pH 8.5 in the presence of 5 mM CaCl_2_ (Sigma-Aldrich C1016) in 96-well plates. This resulted in an increase in absorbance at 405 nm, which was monitored on a ThermoScientific Multiskan GO plate reader. Increasing quantities of inhibitor were added simultaneously to wells that were otherwise identical, in order to reduce the protease signal. The amount of protease that was rendered inactive per amount of inhibitor was then calculated by linear regression.

### Inhibition of papain

Papain inhibitory activity was performed according to the method of Pouvreau [20]. Briefly, l-BAPNA was exposed to papain (Applichem A3824) in a 100-mM potassium phosphate buffer of pH 6.5 (Merck 1.05099) containing 300 mM KCl (VWR Rectapur 26759.291), 4 mM EDTA (Sigma-Aldrich 27285), and 16 mM of cysteine (Fluka 30130). The hydrolysis of the substrate was monitored at 405 nm in a 96-well plate with different wells containing increasing quantities of inhibitor. The amount of protease inhibited per amount of sample material was obtained by linear regression.

### Inhibition of MMP-9

Recombinant human proMMP-9 was produced in Sf-9 insect cells and activated by incubation with the catalytic domain of MMP-3 as previously described [51]. Activation of proMMP-9 was confirmed by a band shift of ± 10 kDa on SDS-PAGE, corresponding to the removal of the auto-inhibitory propeptide domain. To test the inhibitory activity of the potato extracts, we measured the degradation of a fluorogenic MMP substrate peptide (2.5 μg/mL OmniMMP, Mca-PLGL-Dpa-AR-NH2, cat. no. BML-P126-0001, Enzo Life Sciences, Farmingdale, NY) by MMP-9 (0.1 nM) in the presence or absence of different concentrations of extract (1000–0.5 μg/mL). As a negative control, we included the known MMP-9 inhibitor SB-3CT (dose-response) and EDTA (100 mM).

### Inhibition of proteases in human neutrophil degranulates

Neutrophils were isolated from fresh blood of healthy donors, via density gradient centrifugation as described [52]. To obtain neutrophil degranulate, neutrophils were suspended in degranulation buffer (120 mM NaCl, 15 mM CaCl_2_, 20 mM Tris/HCl pH 7.5) at a concentration of 10^7^ cells/mL and degranulation was induced by stimulation with N-formylmethionyl-leucyl-phenylalanine (fMLF) (final concentration 0.5 µM) for 20 min at 37 °C. Next, the supernatant was collected by centrifugation. Proteolysis of an MMP substrate (OmniMMP, broad substrate for most MMP and for ADAM17/TACE) and DQ-elastin by proteases in the neutrophil degranulate (10 µL) was measured in the presence of absence of different concentrations of fractions 1–5, EDTA (a metalloprotease inhibitor), and AEBSF (a serine protease inhibitor).

### Vero E6 cell line for virus infection assays

The African green monkey Vero E6 cell line (ATCC CRL-1586) was maintained in Dulbecco’s minimal essential medium (DMEM) (Gibco), high glucose supplemented with 10% fetal bovine serum (Life Science Production), penicillin (100 U/mL), and streptomycin (100 U/mL) (Gibco). Vero E6 cells were mycoplasma negative and maintained at 37 °C under 5% CO_2_.

### SARS-CoV-2 production and characterization

SARS-CoV-2 was isolated from an infected patient via a throat swab. Sequence analysis revealed that the isolated strain is an alpha SARS-CoV-2 variant and is mycoplasm-negative. The original stock was passaged twice in Vero E6 cells to obtain a working stock. Infectious virus titers were determined by plaque assay on Vero E6 cells and defined as the number of plaque forming units (PFU) per mL. The detection limit of the assay is 150 FU/mL [25].

### Cytotoxicity assay

Vero E6 cells were seeded into 96-well plates at a density of 2 × 10^4^ cells per well. The following day, cells were exposed to increasing concentrations of PI fractions for 19 h at 37 °C under 5% CO_2_. PI fractions were diluted in cell culture medium. Subsequently, cellular cytotoxicity was evaluated using the CellTiter 96^®^ AQueous One Solution Cell Proliferation Assay kit using manufacturer’s instructions from Promega (Madison, WI). Briefly, at 19 h post-treatment, 20 µL of MTS/PMS solution was added per well and incubated for 2 h at 37 °C. Subsequently, 10% SDS was added to each well (2% end concentration) to stop the reaction and the absorbance was measured at 490 nm with a microplate reader.

### Antiviral assay in Vero E6 cells

Vero E6 cells were seeded at a density of 1.3 × 10^5^ cells/well into 12-well plates. The following day, cells were infected with SARS-CoV-2 at a multiplicity of infection (MOI) 1 in presence of increasing concentrations of PI. Infection was done in 250 µL DMEM (2% FBS) medium. At 2 hpi, the virus inoculum was removed, cells were washed twice with plain DMEM medium and fresh DMEM with 10% FBS, and PI were added after which incubation was continued for 6 h. Finally, the cell supernatant was collected and centrifuged to clear from cell debris and the viral titer was determined using plaque assay. Resveratrol was used as positive control in reducing virus transduction; its vehicle (Ethanol) was an additional control.

### Vero E6 cell based ACE2 enzymatic activity assay

Vero E6 cells were plated at a density of 10 × 10^5^ cells into a 96-well plate and incubated overnight at 37 °C, in a humidified 5% CO_2_ atmosphere in DMEM (Lonza, Basel, Switzerland) supplemented with 10% fetal calf serum (FCS) (Sigma Aldrich, F7524, St. Louis, MO, USA). Concentrations between 100 nM and 1 μM were used for fractions 1–5 and incubated for 6 h at 37 °C, whereafter 2 µL of the ACE2 protease substrate solution (Abcam, UK #111,750) was added. *Ex*/*Em* was 485/530 nm for the measurement of the proteolysis.

### Proteomics analysis

Fractions 1, 2, 3, and 4 were selected for proteomics analysis. Protein samples were loaded on an 8% pre-cast RunBlue gel (Expedeon) and run at 100 V for 5 min. Gel staining was performed using InstantBlue (Expedeon) followed by a wash with ultrapure water. Coomassie-stained bands were excised in one gel slice that was further cut into small pieces and destained using 70% 50 mM NH_4_HCO_3_ and 30% acetonitrile. Reduction was performed using 10 mM DTT dissolved in 50 mM NH_4_HCO_3_ for 30 min at 55 °C. Next, the samples were alkylated using 55 mM chloroacetamide in 50 mM NH_4_HCO_3_ for 30 min at room temperature and protected from light. Subsequently, samples were washed for 10 min with 50 mM NH_4_HCO_3_ and for 15 min with 100% acetonitrile. Remaining fluid was removed, and gel pieces were dried for 15 min at 55 °C. Tryptic digestion was performed by addition of sequencing-grade modified trypsin (Promega; 25 µL of 10 ng/mL in 50 mM NH_4_HCO_3_) and overnight incubation at 37 °C. Peptides were extracted using 5% formic acid in water followed by a second elution with 5% formic acid in 75% acetonitrile. Samples were dried in a SpeedVac centrifuge and dissolved in 20 µL 5% formic acid in water for analysis with LC-MS/MS.

The samples were analyzed on a nanoLC-MS/MS consisting of an Ultimate 3000 LC system (Dionex, Amsterdam, The Netherlands) interfaced with a Q-Exactive plus mass spectrometer (Thermo Fisher Scientific). Peptide mixtures were loaded onto a 5 mm × 300 μm i.d. C18 PepMAP100 trapping column with water with 0.1% formic acid at 20 μL/min. After loading and washing for 3 min, peptides were eluted onto a 15-cm × 75-μm i.d. C18 PepMAP100 nanocolumn (Dionex). A mobile phase gradient at a flow rate of 300 nL/min and with a total run time 75 min was used: 2–30% of solvent B in 87 min; 30–80% B in 5 min; 90% B during 1 min, and back to 2% B in 0.1 min. Solvent A was 100:0 water/acetonitrile (v/v) with 0.1% formic acid, and solvent B was 0:100 water/acetonitrile (v/v) with 0.1% formic acid. In the nanospray source, a stainless-steel emitter (Thermo Fisher Scientific) was used at a spray voltage of 2 kV with no sheath or auxiliary gas flow. The ion transfer tube temperature was 250 °C. Spectra were acquired in data-dependent mode with a survey scan at m/z 300–1650 at a resolution of 70.000 followed by MS/MS fragmentation of the top 10 precursor ions at a resolution of 17,500. Singly charged ions were excluded from MS/MS experiments, and fragmented precursor ions were dynamically excluded for 20 s.

The PEAKS Studio version Xpro (Bioinformatics Solutions, Inc., Waterloo, Canada) software was used to search the MS data against a protein sequence database of potato Solanum tuberosum [53] derived from the work of Xu et al. [54]. Search parameters include trypsin digestion with up to two missed cleavages, fixed modification carbamidomethylation of cysteine, variable modification oxidation of methionine, precursor mass tolerance of 20 ppm, and fragment mass tolerance of 0.02 Da. The false discovery rate was set at 0.1% on the peptide level.

### Supplementary Information

Below is the link to the electronic supplementary material.

Supplementary file1 (DOCX 16 KB)
