# Supplementary material for: Novel high-yield potato protease inhibitor panels block a wide array of proteases involved in viral infection and crucial tissue damage
Source: J Mol Med (Berl). 2024 Feb 21;102(4):521–36. doi: 10.1007/s00109-024-02423-x (PMC10963447; doi:10.1007/s00109-024-02423-x)
Supplement: Supplementary file 1 — Supplementary file1 (DOCX 16 KB) [file 109_2024_2423_MOESM1_ESM.docx]

**Supplementary Material**

**Table S1:** UniProtKB/Swiss-Pro accession numbers of protease inhibitors identified in the fractions.

| *Accession* *number* | *Protein* |
| --- | --- |
| Q41480 | Aspartic protease inhibitor 1 |
| Q43646 | Aspartic protease inhibitor 2 |
| P58518 | Aspartic protease inhibitor 3 |
| Q43645 | Aspartic protease inhibitor 4 |
| P58519 | Aspartic protease inhibitor 5 |
| P58520 | Aspartic protease inhibitor 6 |
| Q41448 | Aspartic protease inhibitor 7 |
| P17979 | Aspartic protease inhibitor 8 |
| P58521 | Aspartic protease inhibitor 9 |
| Q03197 | Aspartic protease inhibitor 10 |
| P16348 | Aspartic protease inhibitor 11 |
|  |  |
| P20347 | Cysteine protease inhibitor 1 |
| P24744 | Cysteine protease inhibitor 2 |
| O24388 | Cysteine protease inhibitor 3 |
| P58602 | Cysteine protease inhibitor 4 |
| O24385 | Cysteine protease inhibitor 7 |
| O24384 | Cysteine protease inhibitor 8 |
| Q00652 | Cysteine protease inhibitor 9 |
| O24383 | Cysteine protease inhibitor 10 |
|  |  |
| P58514 | Serine protease inhibitor 1 |
| P58515 | Serine protease inhibitor 2 |
| P58517 | Serine protease inhibitor 4* |
| Q41484 | Serine protease inhibitor 5 |
| P30941 | Serine protease inhibitor 7 |
| P24743 | Serine protease inhibitor 8 |
| Q41433 | Probable serine protease inhibitor 6 |
| P01052 | Chymotrypsin inhibitor I A B and C* |
|  |  |
| Q00783 | Proteinase inhibitor 1* |
| P01079 | Proteinase inhibitor PTI |
| Q00782 | Proteinase inhibitor type-2 |
| Q41435 | Proteinase inhibitor type-2 T |
| P01080 | Proteinase inhibitor type-2 K |
| Q43652 | Proteinase inhibitor type-2 CM7 |
| Q41488 | Proteinase inhibitor type-2 P303.51 |
| P08454 | Wound-induced proteinase inhibitor 1* |
|  |  |
| P01075 | Metallocarboxypeptidase inhibitor* |
| P37842 | Multicystatin* |
|  |  |

* indicates protease inhibitors with relative abundance lower than 1.2% in all 4 fractions
